# Influence of Sleep Monitoring Terminal Displays-Related Factors on User Visual Perception via Eye-Tracking and Tasks

**DOI:** 10.3390/jemr19040077

**Published:** 2026-07-14

**Authors:** Jianghao Xiao, Jinyi Zhi, Shiyu Dong, Yao Zhou, Yao Xiao, Dengkai Chen

**Affiliations:** 1 School of Design, Southwest Jiaotong University, Chengdu 611756, China; 2 School of Mechanical Engineering, Northwestern Polytechnical University, Xi’an 710072, China

**Keywords:** visual perception, pupillometry, user experience, task load, satisfaction

## Abstract

The sleep monitoring terminal display (SMTD) designated for sleep cabins provides data visualizations of sleep profiles, yet poses challenges concerning visually informed interfaces. Given the scarcity of research on the SMTD interface, this study aims to evaluate the influence of SMTD-related factors, including stimulus areas of interest (AOIs), user experiences and tasks, on user visual perceptions while interacting with the SMTD system. Eye-tracking experimental contexts drawn from authentic settings are used to examine how the SMTD interfaces affect visual perceptions under varying tasks. Forty valid samples were collected, and pupil sizes (PSs), task performances, satisfaction, and usability were statistically compared and evaluated. The findings indicated that tasks had no significant effects, but user experiences and stimuli AOIs had significant main effects on PSs. In addition, task completion time ratio and tracking ratio between the two tasks varied; physical demand exceeded mental demand in task 1, whereas it was the opposite in task 2. The effectiveness of post-optimized interfaces was additionally substantiated through combining subjective ratings and objective metrics. The SMTD study provides novel insights for digital interface development and helps enhance users’ integrated visual perception.

## 1. Introduction

According to data released by the Chinese Sleep Research Society, over 300 million people in China have suffered from sleep disorders, while big data in Japan indicates an increase in shorter sleep duration and later bedtime per year accompanied by insomnia and poorer sleep [1]. However, sleep occupies one-third of a person’s lifespan, which affects the remaining two-thirds. Currently, digital health management as a feasible paradigm provides longitudinal sleep information for effective monitoring [2]. The custom-designed sleep monitoring terminal display (SMTD) targeted at napping pods or capsules for different scenarios helps manage sleep profiles with improved health awareness in the absence of essential sleep knowledge among persons. Specifically, the SMTD is displayed as an independent computer screen where various sleep metric data depict their primary features. In the course of human–computer interaction (HCI), the SMTD serves as the primary medium through which the sleep monitoring system within the sleep pod interacts with users.

In a digitally transformed society, displays provide an interface for accessing information and data. When a user interacts with a computer display, more than 80 percent of external information comes via visual channels [3]. Visual perception is a segment of visual cognitive processing [4]. Selective processing highlighted has an interplay with interface-coded information acquisition through the eyes [5,6]. Yet human cognitive capacity is limited, and an unreasonably complex interface design influences human cognitive performance. Cognitive load theory suggests that factors like task complexity and time pressure directly influence the users’ intrinsic sensory load [6]. Traditional measures of visual perception, such as cognitive walkthroughs, heuristic evaluation, and user testing, have offered insight. Nevertheless, cognitive walkthroughs are difficult to engage with real users whose perceptions or cognitions are gained through semantic questions [7,8]. Heuristic evaluations primarily reflect subjective thoughts of experts [9,10,11]. User testing is an examination centered on users or evaluators, yet corresponding assessment criteria need to be established [9]. Compared with traditional measurements, assessments using eye-tracking (ET) technology can directly gauge user visual attention, which is less subject to psychological biases and more reliable.

The user’s eyes are focused on the graphical display panel, frequently checking sleep status through the SMTD. Considering the importance of the SMTD function designed to monitor sleep status, this study is dedicated to enhancing user-centered data visualization and decreasing task load under specific conditions. Nevertheless, few studies have been conducted on visual perceptions for the SMTD interface. The perceived performance of user interaction with the display panel is uncertain and needs to be validated in the face of an emerging display. Following the findings of panel-based information display evaluation studies [12,13], user perception and experience of the display are interconnected in some manner. Based on the above-mentioned content, the SMTD-related elements, user experience, and tasks are assumed to evoke different visual perceptions comprehensively.

Therefore, this study aimed to evaluate the influence of the SMTD factors, including display stimuli, user experiences, and tasks, on measured metric variables corresponding to visual perception. This study investigated both objective (ET technology) and subjective (questionnaire survey) methods to obtain visual perception. The pupillary responses, task performance, and subjective ratings were characterized as outcomes to comparatively analyze the effects of the pre- and post-optimization scheme with regard to the SMTD stimuli. This study also examined the integrated effects of post-optimized SMTD in the experimental context and in real-world settings. The research primarily addresses three questions:

RQ1: How do experienced versus inexperienced users interact with and perceive the SMTD interface system?

RQ2: Is there a significant effect on user visual perception when faced with two different tasks?

RQ3: Has the optimized alternative scheme been enhanced based on the integrated evaluation and analysis?

This paper is organized as follows. Section 2 reviews existing related literature, elucidating ET technology and information display, and demonstrating the necessity of this study. Section 3 presents experimental materials and methods. The experimental and statistical analysis results are shown in Section 4. Further discussions are presented in Section 5, probing both practical and theoretical significance, and noting the limitations of the research. Section 6 concludes the paper.

## 2. Related Work

To understand user visual perception when interacting with real-world interfaces, it is necessary to utilize ET technology and clarify ET metric characteristics through examples of existing applications. When designers conceive alternative display interface design schemes, the way to enable users to effectively acquire interface information in specific contexts is the starting point for the research, and meanwhile, user background factors need to be explored besides traditional display interface elements.

### 2.1. Eye Tracking Technology

The ET technology provides us with a way to understand the distribution of attention and user perception while navigating through panel-based information displays, such as industrial computers, tablet computers, web pages, and mobile application software (App) [9,14]. An earlier study employed ET data for “good-bad” evaluation of computer interfaces [15]. The evaluation results reported the interface differences in users’ perception. For extensive HCI research, eye movement data reflect temporal-spatial attention characteristics of displayed information [16]. Recently, some scholars have used ET technology to capture the perceived experience of static and dynamic product network displays [17]. Among ET experiments based on specific tasks, eye movement signals are also applied to the monitoring of computer screen activities and user experience evaluation of ticket vending machines [18,19]. Another study co-created with people living with dementia and caregivers to design a digital App, and further identified App usability issues by ET technology [20]. By reviewing recent research on HCI interfaces with perceptual measures, it found that ET technology had a wide range of applications [3,17,18,19,20,21], and the potential for various display terminal interfaces [12,13,15,16,22,23,24,25]. Hence, the intrinsic user visual perception of the SMTD interfaces was investigated via the ET method.

Eye movement metrics are often viewed as objective measurements. Fixation-based (e.g., fixation count and fixation duration) and saccade-based (e.g., saccadic count and time) metrics are the two most common representations, with fixation being the most widely used [26]. Many studies have not considered the usage of pupil size (PS) for characterizing the visual perception of an interface. Within the panel-based information display, the PS metric is often neglected, but can convey a wealth of eye movement information. Particularly enlarged pupil change occurs when viewing emotionally arousing pictures [27]. In practice, a strong link between PS variation and web page user preferences is observed [28]. Another study found a relationship between pupil enlargement and risk perception [29]. Pupillometry was used as a possible pathway for measuring mental workload in the context of a healthcare setting [30,31]. The usage load of health information systems can be reflected by measuring PSs. To sum up, pupil diameter sizes are worth using for studying the effect on visual perception, despite the fact that fixation or saccadic variables were frequently considered in the past [12].

### 2.2. Information Display

Several publications revealed that color and layout were the two most important factors influencing cognitive interfaces or visual display units. An experimental study examined the computer system display when exploring the effect of background color of learning materials on cognitive load [32]. The superimposed foreground and background images increased the complexity of transparent display information and reduced the accuracy of color information [33]. For multicolor user interfaces, Bodrogi studied the conspicuousness experiments of visual perception about colored text based on the theoretical formula of chromaticity contrast [34]. Even more meticulously, how different types of highlighting and text display color combinations affected cognitive (i.e., reading and searching) performance was studied [35]. Overall, it was concluded that the best display was in color. As the foreground brightness was lower than the background, or foreground chroma was lower than the background, the user’s reading speed was faster, but visual perception preference was lower. Additionally, the term usability related to ergonomics of human-system interaction reminds us of the research roadmap from the initial goal, the user, and the context [36]. Given this, the study is mainly aimed at users coupled with user experience in the context of the SMTD system. Therefore, this study delves further into the impact of colorful display interfaces when performing user search tasks.

On the other hand, the effect of ambient illumination on text recognition was explored [37]. This display contrast of black text on a white background could adapt to complex lighting conditions. The effects of illumination and color contrast on information search in a display device were investigated [38]. The results indicated that high luminance contrast between text and background had a good speed of visual perception when searching the user interface. The visual acuity of color differences was much lower than that of luminance differences. However, some studies have shown that text-background polarity affected performance in experiments, independently of ambient lighting and color contrasts [39]. This contradictory view also motivated the experiments on different text-background displays under identical illumination conditions in this study. Previous studies have indicated that past experience served as a determinant of visual form perception with other determinants remaining constant [40]. Whether this phenomenon exists on the SMTD interface remains to be substantiated.

## 3. Materials and Methods

### 3.1. Participants

Forty recruited postgraduate students (half of them female) participated in the experiment. Participants’ ages ranged from 21 to 36, averaging 24.9 ± 3.2 years, and their registered information included sex, age, and corrected eye vision. None of them had color weakness, color blindness, or other physiological abnormalities. Before the experiment, they were also classified into two categories (experienced group [E1, n = 22] vs. inexperienced group [E2, n = 18]) by asking some queried entries to judge, as shown in Table 1. Inexperienced means no prior exposure to or use of equipment for sleep monitoring purposes. Inexperience and experience can be viewed as the two extremes of a single axis. Through the sum of quantification scores for these three questions, inexperienced is defined as a score ranging from 3 to 7, while experienced is set at a score range between 8 and 12. Therefore, if all three questions are answered, 3 is the minimum score, and 12 is the maximum. Groups E1 and E2 were each composed of 10 males. Informed consent was obtained from all involved subjects prior to data collection. All of them voluntarily participated in the study and were rewarded with 7 dollars after the experiment.

To validate the rationality of the grouping, reliability and validity analyses were conducted. The Cronbach’s alpha coefficient was 0.816, indicating that these three items possessed high internal consistency reliability. The KMO coefficient was 0.715, which demonstrated that correlations existed among the items. Bartlett’s sphericity test showed significance (p < 0.001), rejecting the null hypothesis and thus implying that related items were suitable for integration into a common dimension.

### 3.2. Measured Metrics

First of all, the study used the PS variations to measure the pupillary responses with reference to available literature [27,28,29,30,31]. The tracking ratio was employed to signify the accuracy of visual tracking, due to the divergence in individual (pointing to the head and eyeblink movements) and target velocities [41]. Simultaneously, the success rate of answering questions was selected to count the cognitive accuracy and study whether digital display interfaces successfully supported the completion of tasks. Secondly, task load assessment (TLA) was adopted because performing visual behaviors required massive attentional resources. Three construal experiences of self-report revealed psychological modes of self-reflection, namely momentary, retrospective, and prospective reflection [42]. Subjective ratings, including user satisfaction and SMTD system usability, were obtained through momentary and retrospective reflections.

Detailed definitions of these seven measured metrics, which are also dependent variables, are as follows:

1. PS [unit: mm]: The statistical average pupil diameter size of subjects in an area of interest (AOI);
2. Task completion time ratio (TCR) [unit: %]: The ratio of task completion time in Task 2 (T2) to the fixed time in Task 1 (T1);
3. Tracking ratio (TRR) [unit: %]: The accuracy of effective visual tracking for interaction between subjects, display panel, and implicit ET devices, quantifying the quality of data collected during interactions, accounting for unavoidable artifacts such as blinking, horizontal eye movements, and head motion;
4. Success rate of answers to questions (SAQ) [unit: %]: The match ratio of subjects’ choices to actual values displayed in the original interface (1—matching, 0—mismatching). The SAQ formula is presented as Equation (1) in the Data analysis section;
5. TLA: It consists of task difficulty level (TDL) by Single Ease Question [12], and the NASA Task Load Index (NASA-TLX) scale [43]. Single Ease Question denotes “Overall, what is the difficulty level of this task?” using a 7-level Likert-type rating (1—very difficult, 7—very easy), while NASA-TLX scale refers to six dimensions comprising mental demand level (MEL), physical demand level (PHL), temporal demand level (TEL), task performance level (TAL), effort level (EFL), and frustration level (FRL), scoring through a 7-level Likert-type rating (1—very low, 7—very high);
6. Overall satisfaction (OSA): This item of satisfaction questionnaire originated from three existing scales, including usefulness, satisfaction, and ease of use [44], software usability measurement inventory [45], and the post-study system usability questionnaire [12,46], employs a 7-level scale (1—very dissatisfied, 7—very satisfied) enquiring “Overall, whether you are satisfied with working with it”;
7. SUS score: Ten questions made up the System Usability Scale (SUS), adopting a 5-level scale (1—strongly disagree, 5—strongly agree) [10,47]. The SUS was originally developed with the aim of providing a measurement of user perceptions for the usability of a system in 1986, which included ten questions, of which five were positive descriptions (odd items), and five were negative descriptions (even items). The SUS was considered a tool for evaluating efficiency, effectiveness and satisfaction.

In terms of these metrics, objective PS variations play a dominant role, while subjective OSA and SUS scores for display stimulus provide direct feedback from the users. In the SUS score framework, satisfaction is one of the three core components of usability, captured through specific items designed to measure this dimension. The OSA leans toward a comprehensive evaluation, extending beyond the realm of usability to encompass participants’ overall emotional response and acceptance of the system interface. The OSA points to the ultimate outcome of the interaction experience. In parallel, task performance metrics (TCR, TRR, SAQ, TLA) focus on comparing the potential impact of different tasks between user subgroups.

### 3.3. Experimental Stimuli

**Figure 1:**
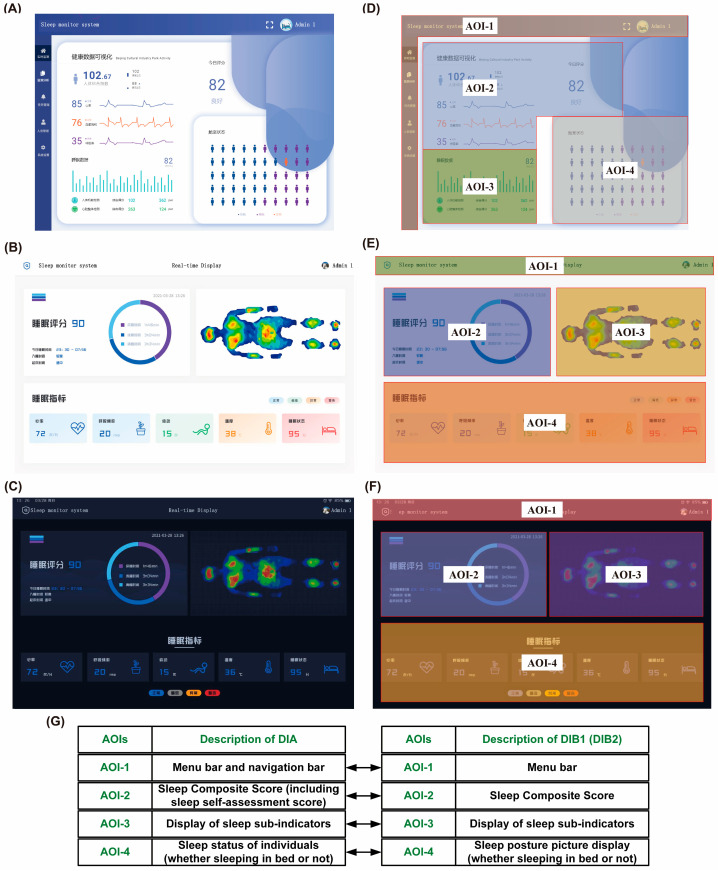
Experimental stimuli of SMTD interfaces referring to (**A**) DIA, (**B**) DIB1, and (**C**) DIB2, segmented AOIs referring to stimuli AOIs of (**D**) DIA, (**E**) DIB1, and (**F**) DIB2, and a description of the four AOIs referring to (**G**) functional information.

Visualized interfaces in the experimental design were the primary stimuli pointing towards the SMTD in Figure 1A–C as stimulus displays. To optimize the display interface, researchers adopted a comprehensive approach guided by design principles focused on enhancing system interface efficiency, improving usability, error prevention, and ease of use. The DIA was the pre-optimization scheme, whereas DIB1 and DIB2 were post-optimizations. The background designs of DIA and DIB1 adopted identical high-brightness colors with distinct colored element layouts. DIB1 in Figure 1B and DIB2 in Figure 1C were assigned to the same layout style corresponding respectively to high and low background brightness. The AOI [48] was an important component of stimuli named and distinguished by semantic information (see Figure 1G). We reasonably divided SMTDs into four AOIs as shown in Figure 1D–F.

### 3.4. Design of Experiment

A mixed-factor design method was applied to the experimental setup. The four-way factors contained: (1) Stimuli interfaces, including three stimuli; (2) AOI, including four AOIs; (3) user type, including the E1 and E2 groups; (4) task type, including T1 and T2. There is a nested relationship between AOIs and the stimuli interface. The experimental task protocol was edited, designed, and presented by E-prime 2.0 software. T1 meant the visual target search and recognition subtask within the display interface, while T2 denoted the numerical comparison subtask. T1 was completed prior to T2. The interval between T1 and T2 was set at 5 min to ensure adequate breaks for these participants. This five-minute break alleviated the fatigue caused by staring at screens, allowing the entire nervous system to relax and minimizing the carryover effects from the prior experiment. Specifically, numerical value selection and comparison with the assistance of short-term working memory were completed in T2. Participants freely searched for the display information they visually deemed most critical in T1.

The display screen randomly presented one of the three SMTDs both in T1 and T2, and then the question selection feedback window appeared alone in T2. Each stimulus interface was repeated three times in each task. Hence, each presentation contained three repeated trials, and therefore two groups of tasks required 3 × 3 × 2 = 18 trials responded by the participants in total. Moreover, we prepared six alternative questions in advance and randomly distributed them to each subject in T2. These questions involved: (1) specific blood oxygen saturation, (2) heart rate, (3) respiration rate, (4) sleep body movements, (5) body temperature, and (6) sleep scores. Each display interface covered at least two questions that closely followed the presentation of the stimuli display interface.

**Figure 2:**
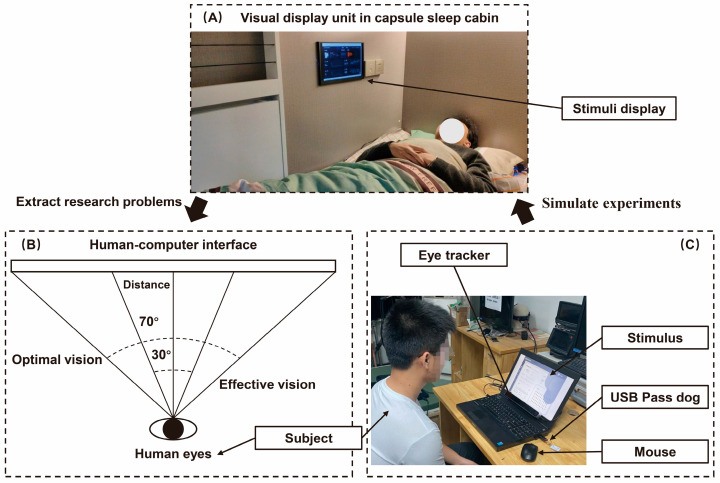
Experimental context of interactions between participants and stimulus interfaces. In real-world scenarios involving (**A**) visual display terminals, research questions regarding (**B**) human-computer interface are identified, and data is collected (**C**) using an eye tracker to conduct simulation experiments.

The interactive context between the subject and the experimental eye tracker is depicted in Figure 2. Following the visual display terminal in a capsule sleep cabin (see Figure 2A), researchers extracted research questions concerning the ergonomic HCI interface in Figure 2B. A telemetry desktop ET system (SMI RED250mobile) cooperated with a notebook computer (system information is Intel(R) Core (TM) i7-4900MQ CPU 2.80 GHz, NVIDIA Quadro K2100M, Microsoft Windows 10 Pro 64-bit) to capture eye-movement data. The eye tracker adopted binocular acquisition, configuring a laptop keyboard and a wireless mouse. The range of head motion was 40 × 40 cm at a control distance of 70 cm. The sampling frequency of the ET device was 60 Hz, and its gaze localization accuracy was <0.5°. The experimental program was compiled using the psychological experiment software E-prime (version 2.0). Concerning the 95th percentile of the human visual field range under standard sitting postures, the visual area within 30° was selected as the central area of vision for the simulated experiment, while the 30° to 70° was visually recognized as the peripheral interference area in Figure 2B. Accordingly, the distance between the two eyes and the displayed screen (or the infrared camera) was limited to 50 to 70 cm to ensure the maximum accuracy of ET experiments. Figure 2C depicts the actual experimental setup designed for experimental simulations, showing the primary eye tracker, the mouse employed, the stimulus, and the USB Pass dog.

This ambient illumination was provided by ordinary indoor light-emitting diodes (LEDs) located at the top, which were mounted 2.7 m above the floor. To eliminate the interference caused by ambiance, an ET experiment was carried out in a quiet lab with constant lighting conditions. Since the ET experiments were performed in a seated position, the illuminance values of ambient lighting at 1.2 m vertical plane eye-level height from the floor were approximately 166.9 lux for this experiment. The correlated color temperature (CCT) of ~5000 K was measured by OHSP-350UV spectral illuminance meter. Other key attributes indicated that color rendering index (CRI) was 79, and the main spectral wavelength was 492.2 nm.

The actual average luminance of the Liquid Crystal Display (LCD, 15.6-inch) display screen during the experiment was 152.4 cd/m^2^, which was measured on a white rendering, while 0.5 cd/m^2^ was measured on a black rendering. For each AOI interface, researchers measured multiple luminance values and performed estimations, yielding the results shown in Table 2. There was no extreme visual contrast between screen and ambient luminance. Images were displayed with a screen resolution of 1920 × 1080 px, refresh rate of 60 Hz, contrast ratio of 50%, color depth of 24 bits, gamma value of 2.2, 100% standard Red Green Blue (sRGB) Color Gamut, and aspect ratio of 16:9 through Lagom LCD Monitor Test Tool and EIZO Monitor Test.

### 3.5. Study Procedure

The whole experimental procedure was divided into three stages: a pre-testing experiment (Stage 1), formal test experiment (Stage 2), and questionnaire experiment (Stage 3). Post-trial questionnaires were launched and collected offline. The details of this procedure are described as follows.

Stage 1: We explained the experimental purpose and process, and its precautions to the subjects, including the collected demographic data of the subjects. We familiarized participants with the experimental procedure. We debugged the ET data acquisition system to make the equipment available. Subjects were required to calibrate the eye tracker under guidance to ensure that the data collected in the experiment was accurate and reliable. Before the beginning, participants’ eyes were calibrated using a 5-point calibration frame.

Stage 2: The subjects read the experimental instructions and then pressed any key on the keyboard to start the formal experiment. A black screen appeared first (presentation time is 1000 ms), and then a black marker appeared in the center of the screen (presentation time is 500 ms). This can be understood as an interval of 1500 ms. Under the instructions, they completed T1 and T2. T1 experiment was carried out first, followed by the T2. In T1, the maximum presentation time of each stimulus was 15 s. When it was over 15 s, the experimental program would automatically jump to the next page. There was no limit to the maximum display time in T2. It actually took about 15 min for a subject to complete T1, and about 5 to 15 min to complete T2. The time spent on calibration and recalibration tasks was allocated to two separate tasks. After each selection in T2, participants must click with the mouse to proceed with the subsequent experiment.

Stage 3: Participants filled in the task-specific load assessment after completing the ET test on stimuli. The two different task objectives theoretically resulted in differences in attentional loads. Task load was assessed separately for T1 and T2. If necessary, the subjects could review these SMTD schemes to continue the usability system evaluation. The questionnaire assessments were performed separately for DIA and DIB.

### 3.6. Data Analysis

This study exported raw eye-movement data from the BeGaze software (version 3.7) of the iViewX eye tracker (manufactured by SMI (SensoMotoric Instruments GmbH), located in Teltow, Germany) and analyzed it by researchers. Because of repeated measures and the fact that the dependent variables conformed to the approximate normal distribution test, multilevel linear mixed-effects models were used to obtain the statistical differences. Compared to traditional linear regression or analysis of variance (ANOVA), the key advantage of mixed-effects models lies in their allowance for intra-group correlation in the data, as it does not require all observations to be completely independent. In this mixed-effects model, user type, task type, stimuli interfaces and AOI were treated as fixed-effects factors and covariates, with the type III sum of squares used to estimate the intercept. Specifically, by treating these participant identification numbers as subjects, the model incorporates a random effect. Random effects are employed to describe the variation in the data caused by random factors, which are typically of no interest to researchers but must be controlled for in the model. Thus, random effects in this case allowed for the uniqueness of individual participants to be taken into account, simultaneously incorporating the covariance type of variance components and associated intercepts into the model.

Participants provided repeated observations across tasks, interfaces, and AOIs. Therefore, analyses of pupillary responses adopted a framework with four factors (User type × Task type × Stimuli interfaces × AOI). Given the nested relationship between the stimuli interface and AOIs, the interaction item Stimuli interfaces × AOI in the mixed-effects model was excluded from modeling analysis.

To further compare variables that had significant effects, the Bonferroni post hoc test method was chosen when the data collected meet the homogeneity of variance (p > 0.05), otherwise a non-parametric test method for non-normal data (e.g., Tamhane’s post hoc test). By inspecting the adjusted means and 95% confidence intervals for different groups, we compared the consistency of the marginal mean estimates with the results of the fixed-effects test. For T2, we calculated the SAQ metric via Equation (1). Due to the huge amount of data, we completed data preprocessing and analysis in SPSS 27.0, and data drawing with Origin 2021. At the same time, the TCR and TRR metrics employed specific two-way models, respectively.

(1) $$SAQ=\frac{3\times N-Mv-Ina}{3\times N-Mv}$$

where N denotes the number of individuals included in the test group, Mv denotes the number of missing values, Ina denotes the number of incorrect answers.

In addition, a papered version of the questionnaire was obtained and converted into electronic data. These collated data were distributed normally, which also accorded with the homogeneity of variance test. Because of the groupings by task and user type, we used a paired sample *t*-test model to compare and verify the results of TLA, OSA, and SUS scores. Descriptive statistics for subjective assessments were accomplished by calculating mean values coupled with interquartile range (IQR), and thus the numerical differences in subscales were graphically represented, since the TLA contained seven dimensions. Importantly, the OSA was measured through satisfaction, whereas the SUS scores were measured through agreement dimensions. Ten questions were converted to SUS scores in steps of 2.5 increments via Equation (2). The SUS score ranged from 0 to 100, in which scores above 68 points indicate a satisfactory level [49].

(2) $$SUS=2.5\times \left(20+\sum S_{Odd}-\sum S_{Even}\right)$$

where SUS denotes the SUS score, $S_{Odd}$ denotes an odd-numbered score, $S_{Even}$ denotes an even-numbered score.

Effect size was an important psychological indicator for comparing the significance of results in empirical experiments [50]. The magnitude of difference between two groups was interpreted as per effect sizes and significance. The *t*-test used Cohen’s $d_{s}$ to calculate effect size (see Equation (3)), whereas ANOVA used partial eta squared ($\eta_{p}^{2}$, see Equation (4)). For example, a large $\eta_{p}^{2}$ effect size requires “>0.14”, while a middle $\eta_{p}^{2}$ effect size requires “>0.06”.

(3) $$d_{s}=\left(M_{1}-M_{2}\right)/\sqrt{\frac{\left(N_{1}-1\right)SD_{1}^{2}+\left(N_{2}-1\right)SD_{2}^{2}}{N_{1}+N_{2}-2}}$$

(4) $$\eta_{p}^{2}=\frac{F\times Df_{Between}}{F\times Df_{Between}+Df_{Within}}$$

where $M_{1}$ and $M_{2}$ represent the mean value of different groups, $SD_{1}$ and $SD_{2}$ represent the standard deviation of different groups, $N_{1}$ and $N_{2}$ represent the number of individuals in different groups; F is the F-value calculated in statistics, Df is the degree of freedom.

## 4. Results

This section focused on the statistical results of pupillary responses and the results of task-related performances regarding TCR, TRR, SAQ, and TLA, as well as depicted the results of subjective ratings regarding OSA and SUS scores.

### 4.1. Pupillary Responses

**Figure 3:**
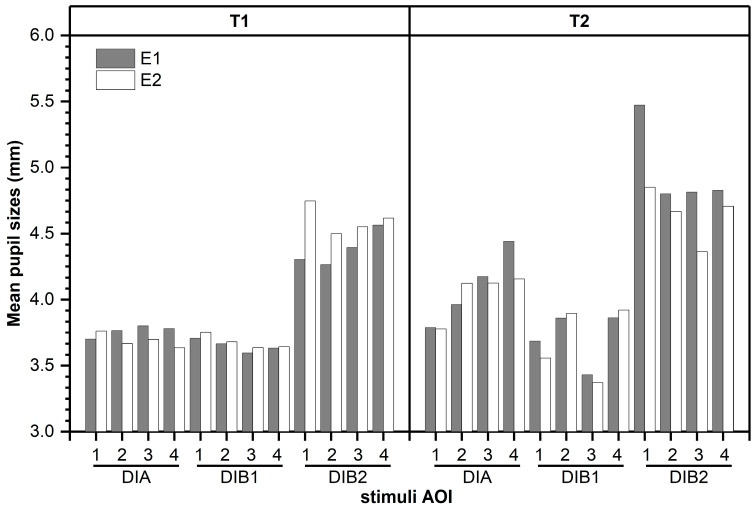
Statistical results of the PS metric.

By comparing statistical effects between different factors as shown in Table 3 and the characteristics of data variation in Figure 3, the PS metric was largely associated with holistic user visual perception and was significant (p < 0.001). This ET experiment utilized black screens and fixation markers as transition phases. The researchers analyzed the transition screen displayed before the stimulus to determine baseline values for pupillary ET metrics, and selected the average pupil diameter as the baseline. Current statistical data indicated that the baseline pupil diameter was 3.2 mm on average, which was lower than the pupil diameters measured in all AOIs. Therefore, the PS metric was used directly for subsequent analysis.

The main effect of Stimuli interfaces on the PS metric was significantly influential (p < 0.001). Simultaneously, there were no statistically significant variations among the different AOIs (p = 0.164). The three-way interaction effect of User type × Task type × Stimuli interfaces was significant (p = 0.001). The tasks in different groups did not have any impact on the PS metric. Paired comparison results in tasks mapped similar features when display information was searched for and recognized visually on the three SMTD interfaces. However, the two-way interaction effect of Task type × Stimuli interfaces was significant (p = 0.001). It was estimated that users’ PSs showed variations across different interfaces under varying tasks. We further summarized the comparative significance of Stimuli interfaces and AOIs between DIA and DIB in Table A1 of Appendix A. A few subjects dwelled at AOI-1 of DIA and DIB1 in the E1 group at T2, and hardly any subjects at AOI-1 of DIB1 in the E2 group at T1. In summary, the effect of the user type factor was significant (p = 0.038). A detailed comparison of PS results for different independent variables is shown below.

#### 4.1.1. Paired Comparisons in Stimuli Interfaces

The PS metrics for Stimuli interfaces were significant. For the comparison of paired stimuli DIA vs. DIB2, or DIB1 vs. DIB2, the significance of PS was shown for mean difference (p < 0.001), which assisted in distinguishing different stimulus AOIs. Stimuli DIA was also clearly distinguished from DIB1 (p = 0.017). The low-brightness background (luminance contrast) and layout style of DIB2 were distinguishable from those of the other two. Under time pressure of tasks, this significant difference in Stimuli interfaces indicated that DIB2 was the most prominent. However, it was difficult for the PS metric to distinguish the perceptive discrepancy between DIA and DIB1, regardless of different users or task factors.

#### 4.1.2. Paired Comparisons in AOIs

For the comparison of four AOIs, notable significances of the PS metric were observed between AOI-1 and AOI-4 (p = 0.037). AOI-1 indicated the function of the menu bar, while AOI-4 displayed information about sleep status or posture. Other pairs of AOIs demonstrated no statistical significance, as shown in Table A1 of Appendix A. The AOI-3 for DIB1 and DIB2 were fully image-based displays, while AOI-4 for DIB1 and DIB2 were a mixture of numbers and text. From a visual cognitive perspective, AOI-1 had a smaller PS than the remaining three AOIs; the PS values for AOI-2 and AOI-3 are close; AOI-4 has the largest PS magnitude. Upon closer investigation, AOI-1 is located above the display interface, while AOI-4 is located below it.

### 4.2. Task-Related Performances

#### 4.2.1. Results of TCR, TRR, and SAQ Assessments

**Figure 4:**
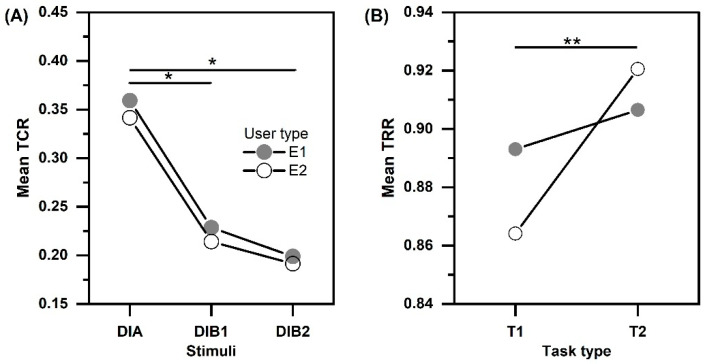
Comparisons of the mean results for (**A**) TCR and (**B**) TRR metrics. Note: Asterisks indicate the significance of effects where * p < 0.05, ** p < 0.01.

T1 was set to a fixed stimulus presentation time, while T2 was determined by the actual perceptive response. Except for one outlier eliminated (TCR = 7.81), the maximum TCR was 1.19. By comparing the mean TCR, DIA was significantly larger than DIB1 and DIB2 in Figure 4A. TCR had a significant effect at the stimulus interface level (F = 3.34, p = 0.039, $\eta_{p}^{2}$ = 0.055 [>0.01]) as shown in Table 4. The outcomes indicated that DIA involved spending more time on visual search and perception, while DIB2 enabled users to find answers to the questions more quickly. In T1, the mean TRR of E1 and E2 were 0.89 and 0.86, respectively. By contrast, the TRR of E1 and E2 were 0.91 and 0.92 in T2, respectively. The TRR at the user type level was insignificant, and the TRR between T1 and T2 was significant (F = 7.18, p = 0.008, $\eta_{p}^{2}$ = 0.016 [>0.01]), as shown in Figure 4B and Table 4. Using the questionnaire results and calculation analysis in Equation (1), the SAQ of the E1 group was 94.28% at T2, while that of E2 was 96.87%. No significant difference in the SAQ scores was observed between the two user groups.

#### 4.2.2. Results of TLA

**Figure 5:**
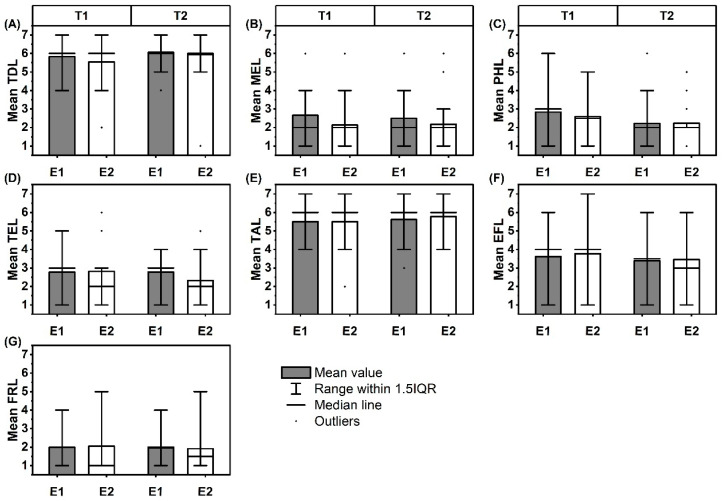
Bar charts for TLA results, including (**A**) TDL, (**B**) MEL, (**C**) PHL, (**D**) TEL, (**E**) TAL, (**F**) EFL, and (**G**) FRL.

These task-related $d_{s}$ effect sizes were all above 0.80, as shown in Table A2 of Appendix A, suggesting a significant impact on task load. Further, the results for seven TLA subscales were insignificant in Figure 5 (p > 0.05), indicating that all users had no extreme difficulties in performing either task. Through synthesized analysis, the EFL had the greatest impact, whereas the TAL was the least impacted. It was inferred that users felt more physical than mental load in T1, yet the opposite phenomenon occurred in T2, as T2 required users to answer cognitive questions. The impact of MEL was ranked before PHL, while the TDL was in the middle of the entire ranking. No significant difference existed between E1 and E2.

Generally, T2 was more difficult than T1, but both were classified as “very easy,” which is consistent with the initial design of the experiment. The objective requirements of the tasks themselves are reflected in mental, physical, and temporal demands, while the other three metrics (TAL, EFL, and FRL) focus on evaluating participants’ subjective reactions and the effort expended during interaction with the system interface. Therefore, even when participants are satisfied with their achievement of the goals, they may still experience a certain degree of frustration and negative emotions, as shown in Figure 5. This suggests that the cognitive load stems from two dimensions: the demands of assigned tasks and the interactive experience with the system interface.

### 4.3. Subjective Ratings

**Figure 6:**
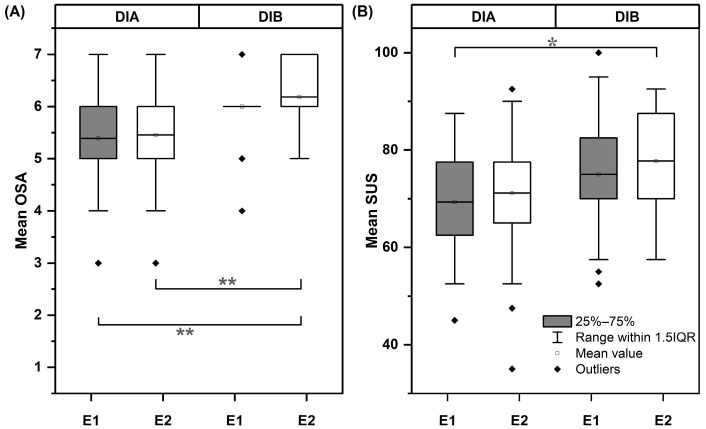
Statistical results of subjective ratings: (**A**) OSA and (**B**) SUS score. Note: Asterisks indicate the significance of effects where * p < 0.05, ** p < 0.01.

The mean OSA in Figure 6 was all “satisfied” (score = >5). The OSA had a significant difference between E1 in DIA and E2 in DIB (t = −2.83, p = 0.007), and meanwhile, the discrepancy of E2 between DIA and DIB (t = −2.92, p= 0.006) was significant. There was a significant difference between E2 in DIB and E1 in DIA (t = −2.44, p = 0.019) for the SUS score. The mean SUS and OSA of E2 in DIB were generally larger than those of other groupings. Inexperienced users were more likely to express satisfaction with the aid of the SMTD visualization.

The mean OSA indicated that these participants had more positive emotional responses to and higher acceptance of the DIB sleep monitoring system. SUS scores did not fully align with OSA outcomes. For the inexperienced group (E2), OSA ratings were more effective in quantifying differences between the pre- and post-optimization schemes, whereas SUS scores showed no significant distinction.

## 5. Discussion

This study evaluated the effects of SMTD-related factors (display interface itself, experienced or inexperienced users, and task type) on visual perceptions using ET technologies, task-related performance, and subjective ratings. The post-optimized scheme (stimuli DIB2 with low background brightness) regarding the outcomes of the PS metric, task performance assessment, subjective satisfaction, and SUS score was comprehensively verified to be optimal.

### 5.1. Eye Movements in Relation to Display

ET was widely adopted in the realm of display research because eye movements could reflect visual search patterns, which were important for revealing cognitive processing mechanisms [24,51]. The present study derived significant effects of stimuli AOI and task type on the PS metric, indicating both main and interaction effects. To fully understand the interaction effect of stimuli AOI and task type, this study conducted a main effect analysis of stimuli interfaces and AOI; thus, it obtained multiple pairwise comparisons of PS metrics between stimuli AOIs. Based on the results available in this study, the DIB2 interface was significantly differentiated from the other two regardless of the type of task. There was difficulty in distinguishing between DIA and DIB1 stimuli using the PS metric in both tasks. This could be explained by identical high background brightness (display with low luminance contrast) delivering a similar user experience of the display interface in the absence of particularly large amounts of text [45].

Essentially, significant PS changes were observed when confronted with different interfaces and the same task. The visual search task was a complex activity associated with reading graphical information through the eyes. An early study showed that the process of graph comprehension was modeled as encoding, pattern interpretation, and integration stages [52]. However, it was difficult for researchers to capture subtle changes in the visuals of user-display interactions. Exploring the effects of digital SMTD interfaces on user visual perceptions required image-like stimuli as input, which depended on the display technology. ET is a convenient tool for capturing visual attention and for supporting the understanding of cognitive phasing when visual content is being viewed. By analyzing valid eye movement data within a given AOI, the majority of participants’ visual attention was spent searching for sleep monitoring information, rather than appreciating or looking at lines associated with the display interface design. Therefore, visually perceptual differences in stimulus displays composed of the background brightness and layout style were proven to be validly characterized by the mean distribution features of the PS metric, when compared to the baseline value in the task-free stimuli interface.

Furthermore, the pupillary characteristics seemed to indicate that the goal of the experimental task in E1 was more targeted than that in E2 for these tasks. Display-based ET experiments and pupillary dilation analysis were combined to identify key objects of website pages [53]. Pupil dilation correlated with the perception of object preferences on a web page. Similarly, the relatively larger results of mean PSs for the DIB2 interface suggested the occurrence of pupillary dilation compared with baseline values. It indicated that the post-optimization (DIB) was clearly distinct from the pre-optimization (DIA) in Figure 6. Coupled with the TCR outcome in Figure 4A and PSs in Figure 3, the low background brightness of DIB2 reflected the highest satisfaction and higher agreement with usability. Given this, DIB2 is considered to be closer to the desired goal of optimizing the display interface.

In particular, the practical display unit is inseparable from the illumination, contrast and interface layouts (segmented AOI) of the content [54]. The SMTD provided access to the sleep status of users within the sleep capsule cabin. The low ambient brightness further accentuated the displayed text and numbers on a low-brightness background inside a dark cabin at night, compared to a bright daytime environment during the day. Meanwhile, a common screen resolution and displayed text were currently applied on a 15.6-inch display, and changing these parameters might alter users’ visual perception features in this study. However, this contrast is limited for existing display technologies. LCD and Organic Light-Emitting Diode (OLED) boast an advantage, due to thin physical panels and clear, stable color rendering. Vacuum Fluorescent Display (VFD) needs to be shaded to prevent glare when operated at night, which is not well-suited for in-cabin sleep monitoring applications.

### 5.2. Users, Tasks, and Visual Perceptions

This study examined both experienced and inexperienced users. By contrast, the presence or absence of experience with wearable devices was investigated as an important variable in the selection of subjects [55], but it was still biased in favor of the experienced ones.

Subsequently, no significant difference between first-time users and those who had experience of a telemedicine service was observed [56], which was somewhat different from the present study. This study revealed that past relevant experiences of users had a similar significant impact on the visual perception between three stimulus display interfaces, which was expected based on previous research [40]. This proximity in results was probably due to the fact that the letter-form figures used also fall under the category of visual perception consistent with this study.

In addition, inexperienced users are more inclined to be satisfied with the visual perception in the present study. Accordingly, this study proposes several possible explanations for this finding. First, this definition of the user type was decisive in Section 3.1 of this study, favoring a cumulative experience of use, with regard to similar sleep health-related monitoring systems or relevant devices for the provision of services. Compared with existing research [56], inexperienced users corresponded to first-time users, while the remaining group was assigned to experienced users. Secondly, the three SMTD interfaces designed in this study could not be associated with the term interface complexity, concerning the number of elements and the density of interface elements. Lastly, when exposed to new sleep monitoring applications at the early stage, researchers or designers tended to maintain a positive user experience and, meanwhile, avoid frustration with their efficacy and efficiency [55].

Although all subscales of the TLA were not significant, it was found that the two tasks required divergent types of loads. The existing cognitive load theory implied that goal-directed visual perception was affected by the task type and partially attracted by time pressure [6]. However, salient information features in the SMTD interface were verified to attract subjects’ visual perceptive attention during information retrieval based on eye movements. The low-brightness background of DIB2 highlighted sleep-monitoring data information that forced subjects to be extremely concentrated and thereby completed the task faster. This outcome in this study was consistent with an existing study [38], indicating that white text on a dark background yielded a higher search efficiency.

The display interface type affected its usability and made visual behavior across AOIs observable beyond the allocation of attentional resources. Bangor et al. compared mean SUS scores for six interface types [49]. The mean SUS score in this study was above 70 points, and it could reach a maximum of 100. This result was plausible in comparison with other publications [49,55]. Faced with similar interface design and evaluation for sleep-monitoring systems, the researchers recommended the SUS as a priority, because the SUS score had a high degree of distinction. Such an inspection extended to display usability was encouraged throughout the development life cycle of health information systems [57]. The SAQ reaching above 90% suggested a high reliability upon completion of the task. Through synthesis, the evaluated system interface usability of DIB in E2 was concluded to be the best; users no longer needed guidance on the menu bar without being noticed in T2. The “sleep score” area should still be placed in the upper left corner and be the most visible display region based on the priority layout principle. The subsequent iterations of the SMTD should begin with the stimuli DIB design proposal.

Taken together, this study achieved a combination of subjective and objective methods to evaluate visual perceptions of the interface displayed by a simulated ET experiment. Some similar cases had also shown that this combined paradigm was the future trend [12,13,22,23], and was one of the perceptive process types postulated by the eye-mind extendedly [29]. Some studies only adopted ET measurements to convey numerical meaning [24,58], whereas subjective self-reports helped dig deeply into findings from display panels.

### 5.3. Limitations

However, despite the above strengths, this study had several limitations. First, this study investigated a limited set of experimental metrics, yet many other latent metrics could characterize users’ visual perceptions. Due to technical equipment factors, some eye movement events cannot be accurately identified and classified. The defined AOIs and shapes were fixed rectangles. AOI-based segmentation methods could have an impact on the clustering of visual attention within the display interface.

Second, this paper cannot ignore the potential impact of different age groups. These studied results may not have been fully validated by possible users, such as the elderly, and thus future studies could account for different types of user engagement. Failing to consider the population with visual impairment or eye disease was also a concern, and this population might be unsuitable for using an eye tracker, though they also had potential needs for sleep monitoring management. Since T1 always precedes T2, the impact of task order remains unknown. The combined effects of factors such as task order, practice, fatigue, or habituation will be examined in future studies.

Finally, a previous study had conducted both pre-test and post-test assessments with subjective questionnaires [29], but current subjective ratings were employed merely at the end of testing. In addition to the SMTD interface factors, environmental variables may affect users’ visual perception. However, this study executed experiments where the display was simulated under constant illumination, which was at a gap from the actual complex scenario. Therefore, a reasonable mixture of various actual ambiances, luminance contrast, and display interfaces serving a digital experience will be evaluated in future research.

## 6. Conclusions

In this paper, we conducted an exploratory study of pupillary response, task performance, and subjective ratings applied to evaluate the effects of SMTD factors on user visual perception. This study analyzed and found that prior experiences of users had a significant impact on visual perceptions among three displayed stimulus interfaces. The effectiveness of pupillometry was proven to be feasible when performing different tasks (T1 vs. T2). Task load performance indicated an insignificant discrepancy between the two tasks. Due to various factors inherent to the experimental setting, pupillary responses can, strictly speaking, only serve as an auxiliary indicator, particularly when addressing RQ3 in this paper. In future research, the relationship between pupillometry (or pupillary dilation) and photometric or workload effects warrants further investigation through deepened psychological research paradigms.

Research objectives pointing toward the interaction between users and the emerging SMTD configured with different digital interfaces were implemented in the context of simulated ET experiments. With the improvement of people’s health awareness, the enhanced implication of sleep monitoring and management services is to help users understand their sleep health level. These findings provide valuable references for research into the actual usage of SMTD interfaces. In terms of user interface development, it offers insights for interface design and evaluation teams concerning integrated visual perception. Combining subjective evaluations and objective evidence, the post-optimized DIB2 (low-brightness background) is enhanced in regard to visual perception and satisfaction. In terms of scheme selection, the results of this study help distinguish three displayed interfaces and show direction for subsequent interface iterations.

## Figures and Tables

**Table 1:** Criteria for determining whether experience is present or absent.

Items	Options	Scores
1. How often do you encounter similar sleep monitoring systems in your daily life?	Never	1
	Occasionally	2
	Frequently	3
	Always	4
2. Have you used similar sleep monitoring systems, like wearables or health panels?	Never	1
	1–2 times	2
	3–4 times	3
	5 times or more	4
3. Among all your experiences, you clearly recognize the subtle differences between past experiences and this current one.	This does not describe you at all.	1
	This does not typically describe you.	2
	This typically describes you.	3
	This describes you perfectly.	4

**Table 2:** Detailed average luminance estimates for each AOI per stimulus interface.

Stimuli	AOIs	Estimated Luminance (cd/m^2^)
DIA	AOI-1	147.5
	AOI-2	164.1
	AOI-3	162.7
	AOI-4	149.4
DIB1	AOI-1	164.7
	AOI-2	163.3
	AOI-3	148.2
	AOI-4	150.2
DIB2	AOI-1	0.5
	AOI-2	0.8
	AOI-3	0.9
	AOI-4	0.8

**Table 3:** Statistical effect results for the PS metric.

Variables	*Df*	F	*p*-Value ^a^
Intercept of model	1	31,294.59	<0.001 ***
User type	1	0.22	0.038 *
Task type	1	4.33	0.645
Stimuli interfaces	2	144.83	<0.001 ***
AOI	3	1.71	0.164
User type × Task type	1	0.58	0.448
Task type × Stimuli interfaces	2	3.35	0.035 *
Task type × AOI	3	2.14	0.094
User type × Stimuli interfaces	2	0.15	0.862
User type × AOI	3	4.01	0.008 **
User type × Task type × Stimuli interfaces	2	6.59	0.001 **
User type × Task type × AOI	3	1.77	0.152

^a^ Note: Asterisks indicate the significance of effects where * p < 0.05, ** p < 0.01, *** p < 0.001.

**Table 4:** Statistical effect results for TCR and TRR.

Metric	Variables	*Df*	F	*p*-Value ^a^	$\eta_{p}^{2}$
TCR	Model	5	1.96	0.090	0.079
	User type	1	1.41	0.238	0.012
	Stimuli interfaces	2	3.34	0.039 *	0.055
	User type × Stimuli interfaces	2	1.21	0.302	0.021
TRR	Model	3	3.71	0.012 *	0.024
	User type	1	0.37	0.546	0.001
	Task type	1	7.18	0.008 **	0.016
	User type × Task type	1	2.74	0.098	0.006

^a^ Note: Asterisks indicate the significance of effects where * p < 0.05, ** p < 0.01.

## Data Availability

Data will be made available on request.

## Abbreviations

The following abbreviations are used in this manuscript:

ANOVA	Analysis of variance
AOI	Area of interest
App	Application software
CRI	Color rendering index
CCT	Correlated color temperature
EFL	Effort level
ET	Eye-tracking
FRL	Frustration level
GLM	General linear model
HCI	Human–computer interaction
IQR	Interquartile range
LEDs	Light-emitting diodes
LCD	Liquid Crystal Display
MEL	Mental demand level
NASA-TLX	NASA Task Load Index
OLED	Organic Light-Emitting Diode
OSA	Overall satisfaction
PHL	Physical demand level
PS	Pupil size
SMTD	Sleep monitoring terminal display
sRGB	Standard Red Green Blue
SAQ	Success rate of answers to questions
SUS	System Usability Scale
TLA	Task load assessment
T1	Task 1
T2	Task 2
TCR	Task completion time ratio
TDL	Task difficulty level
TAL	Task performance level
TEL	Temporal demand level
TRR	Tracking ratio
VFD	Vacuum Fluorescent Display

## Appendix A

**Table A1:** Pairwise comparisons of PS metric between different independent variables.

Independent Variables	Compared Variable (I)	Compared Variable (J)	Mean Difference (I–J)	*p*-Value ^a^
Task type	T1	T2	−0.021	0.645
User type	E1	E2	−0.093	0.038 *
Stimuli interfaces	DIA	DIB1	0.118	0.017 *
	DIA	DIB2	−0.645	<0.001 ***
	DIB1	DIB2	−0.762	<0.001 ***
AOIs	AOI-1	AOI-2	−0.108	0.123
	AOI-1	AOI-3	−0.070	0.335
	AOI-1	AOI-4	−0.153	0.037 *
	AOI-2	AOI-3	0.038	0.447
	AOI-2	AOI-4	−0.044	0.382
	AOI-3	AOI-4	−0.083	0.130

^a^ Note: Asterisks indicate the significance of effects where * p < 0.05, *** p < 0.001.

**Table A2:** *t*-test results between task type and user type based on TLA.

Paired [Task—User Type]	Metric	t	*p*-Value	Mean Difference	$d_{s}$ ^b^	Rankings
T1-E1 vs. T1-E2	TDL	0.75	0.460	0.29	1.213	5
	MEL	1.26	0.215	0.53	1.324	4
	PHL	0.55	0.582	0.24	1.375	2
	TEL	−0.09	0.926	−0.04	1.361	3
	TAL	0.00	1.000	0.00	1.100	7
	EFL	−0.35	0.725	−0.16	1.434	1
	FRL	−0.12	0.906	−0.05	1.203	6
T2-E1 vs. T2-E2	TDL	0.27	0.787	0.10	1.169	4
	MEL	0.64	0.523	0.32	1.554	2
	PHL	−0.01	0.990	−0.01	1.267	3
	TEL	1.38	0.176	0.46	1.050	6
	TAL	−0.55	0.584	−0.16	0.920	7
	EFL	−0.12	0.903	−0.07	1.684	1
	FRL	0.10	0.917	0.04	1.061	5
T1-E1 vs. T2-E1	TDL	−0.65	0.518	−0.22	1.021	5
	MEL	0.33	0.744	0.17	1.519	2
	PHL	1.25	0.221	0.61	1.471	3
	TEL	0.00	1.000	0.00	1.215	4
	TAL	−0.35	0.728	−0.11	0.951	7
	EFL	0.43	0.668	0.22	1.539	1
	FRL	0.17	0.862	0.06	0.954	6
T1-E2 vs. T2-E2	TDL	−1.03	0.307	−0.41	1.313	3
	MEL	−0.11	0.913	−0.05	1.379	2
	PHL	1.02	0.315	0.36	1.187	6
	TEL	1.36	0.180	0.50	1.215	5
	TAL	−0.85	0.399	−0.27	1.062	7
	EFL	0.67	0.509	0.32	1.584	1
	FRL	0.36	0.722	0.14	1.261	4
T1-E1 vs. T2-E2	TDL	−0.31	0.758	−0.12	1.230	4
	MEL	1.02	0.315	0.48	1.498	2
	PHL	1.42	0.163	0.61	1.341	3
	TEL	1.26	0.215	0.46	1.146	5
	TAL	−0.96	0.343	−0.27	0.894	7
	EFL	0.31	0.758	0.16	1.587	1
	FRL	0.27	0.791	0.09	1.074	6
T1-E2 vs. T2-E1	TDL	−1.39	0.172	−0.51	1.152	6
	MEL	−0.82	0.415	−0.36	1.387	2
	PHL	0.89	0.379	0.37	1.302	3
	TEL	0.10	0.921	0.04	1.281	4
	TAL	−0.31	0.757	−0.11	1.121	7
	EFL	0.78	0.438	0.38	1.540	1
	FRL	0.27	0.791	0.10	1.191	5

^b^ Note: *t*-test used Cohen’s effect size ($d_{s}$), see Equation (3).
